# The Upper Triassic Polzberg palaeobiota from a marine *Konservat-Lagerstätte* deposited during the Carnian Pluvial Episode in Austria

**DOI:** 10.1038/s41598-021-96052-w

**Published:** 2021-08-17

**Authors:** Alexander Lukeneder, Petra Lukeneder

**Affiliations:** 1 Department of Geology and Palaeontology, Natural History Museum Vienna, Burgring 7, 1010 Vienna, Austria; 2 Department of Palaeontology, University of Vienna, Althanstrasse 14, 1090 Vienna, Austria

**Keywords:** Solid Earth sciences, Palaeontology

## Abstract

A rich assemblage of various marine taxa from the lower Carnian Polzberg *Konservat-Lagerstätte* near Lunz am See (Northern Calcareous Alps, Lower Austria) is described for the first time in detail. The fossiliferous layers were deposited during the Julian 2 Ib (*Austrotrachyceras austriacum* Zone, *Austrotrachyceras minor* biohorizon). The fine-laminated Reingraben Shales comprise abundant and well-preserved members of the marine Carnian food chain. Invertebrates with the bivalve *Halobia*, the ammonite *Austrotrachyceras* and the coleoid *Phragmoteuthis* dominate over vertebrate actinopterygian fishes. Fragile groups such as polychaetes and isopods are entirely preserved as soft body fossils. The diverse assemblage comprises ammonites (*Austrotrachyceras*, *Carnites*, *Sageceras*, *Simonyceras*), coleoids (*Phragmoteuthis*, *Lunzoteuthis*), bivalves (*Halobia*), gastropods (caenogastropods/heterobranchs), one echinoid, thylacocephalan arthropods (*Austriocaris*), crustaceans (the decapod *Platychela* and isopods such as *Obtusotelson*, *Discosalaputium*), polychaetes (*Palaeoaphrodite* sp., eunicid polychaete), acytinopterygians (*Saurichthys*, *Polzbergia, Peltopleurus, Habroichthys*), cartilaginous fishes (*Acrodus*), coelacanth fish (*“Coelacanthus”*), a lungfish (*Tellerodus*), and a conodont cluster (*Mosherella*). Regurgitalites produced by large durophagous fish and coprolites produced by piscivorous actinopterygians accompany the Polzberg palaeobiota along with rare plant remains (*Voltzia*). The entire fauna of Polzberg and the excellent preservation of the specimens present a window into the Upper Triassic assemblage and palaeoenvironment during the so-called Carnian Pluvial Episode (CPE) in the early Mesozoic. The occurrence of the freshwater lungfish *Tellerodus* and the branchiopod *Eustheria,* a member of brackish to freshwater environments, points to the influence of occasional freshwater pulses or sediment transport events on the marine environment. The Polzberg palaeobiota was deposited during the global CPE, triggering the environmental conditions of the Polzberg Basin and resulting in the formation of the Reingraben Shales with the Polzberg *Konservat-Lagerstätte*.

## Introduction

**Figure 1:**
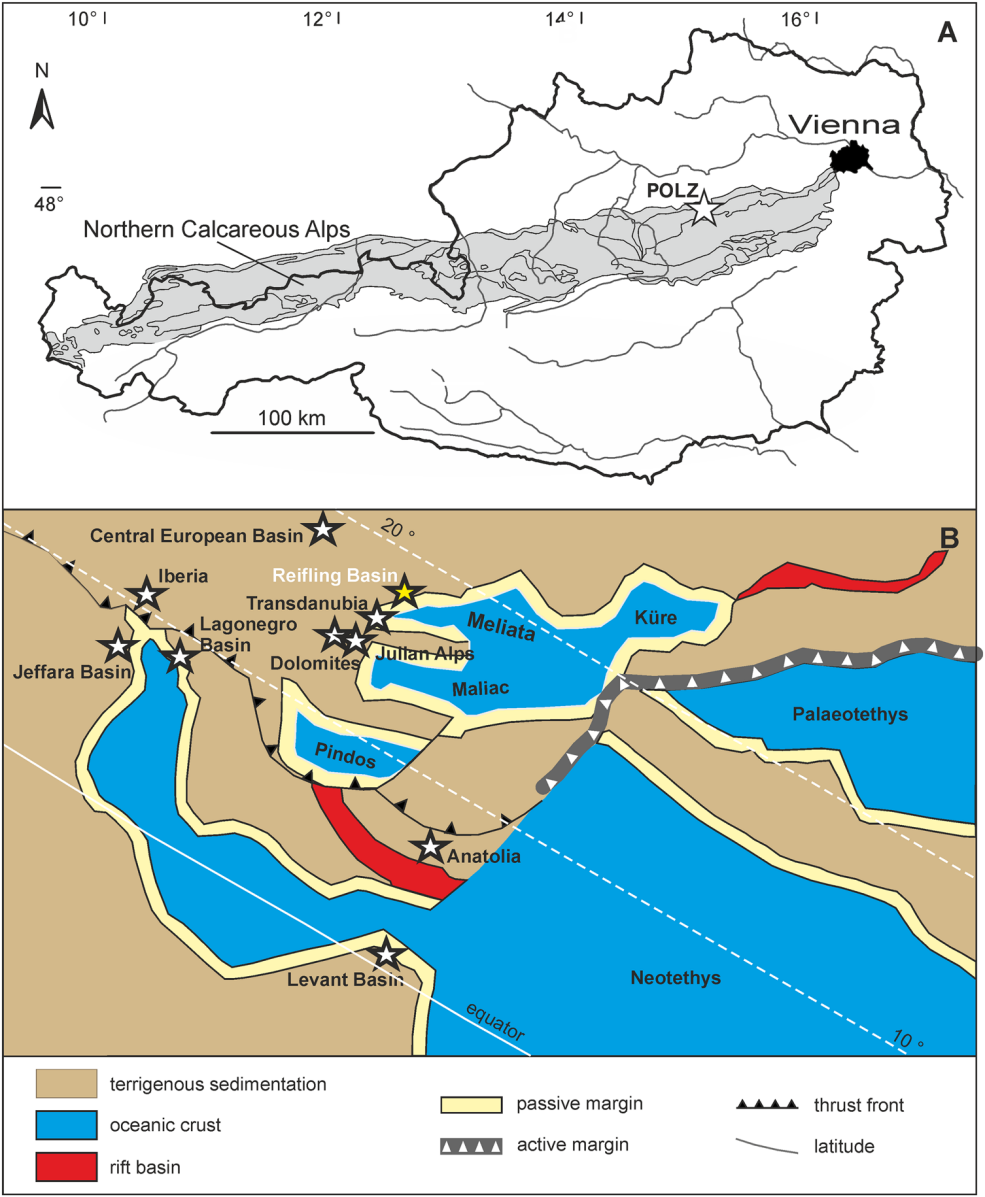
(**A**) Locality map of the Lunz area in Lower Austria and the Austrian Northern Calcareous Alps (in grey). (**B**) Palaeogeography of the Mediterranean region during the Carnian. Asterisk: position of the *Konservat-Lagerstätte* Polzberg (POLZ). Adapted after Lukeneder et al.^19^. Prepared by AL using CorelDRAW X7; www.coreldraw.com.

Palaeobiota of fossiliferous sites known as *Konservat-Lagerstätte*^1^ (see also^2,3^) are precious sources of palaeobiological information^4^. Such conservation *Lagerstätten* provide unique insights into palaeocommunities, food chains and dietary habits, as well as trophic interactions between the inhabitants of marine ecosystems^5^ and references therein. New findings of additional fossil groups and the soft body preservation of numerous organisms shed light on such otherwise hidden Upper Triassic organisms. Middle to Upper Triassic occurrences exhibiting the preservation style of *Konservat-Lagerstätten* are rare^6–8^ and hence even more important for drawing conclusions about palaeoenvironments along with their inhabitants and interactions. The genesis of the Polzberg section is comparable, though not equal in age and taxa, to the Austrian Upper Triassic Seefeld Formation (Norian) fish accumulations from Seefeld (Tyrol)^9^ and Wiestal (Salzburg)^10^. A detailed report on the palaeobiota from the Polzberg area in the Northern Calcareous Alps is lacking so far. Though known for over 140 years, the Polzberg *Konservat-Lagerstätte*, its formation processes and fossil assemblages are poorly understood and new taxa appear almost annually. The locality, situated in Lower Austria (Fig. 1A) and also known as Schindelberg or Pölzberg^11,12^ in historic collections, appears with lower Carnian Reingraben Shales (“Reingrabner Schiefer”, “*Trachyceras* Schiefer”). The unique feature of this *Konservat-Lagerstätte* is the basal few metres, comprising fossiliferous Reingraben Shales with a uniquely preserved fauna^2^. The palaeontological sites in the region of Polzberg are known since the nineteenth century^12,13^. As most of the historical excavation reports and papers were written in German, they failed to reach a broader international scientific community. More recently, new palaeontological data and faunal elements were published from the Polzberg *Lagerstätte*^3,14–18^. The biostratigraphic data hint to a Julian 2 Ib (*Austrotrachyceras austriacum* Zone, *A. minor* biohorizon) age of the main fossiliferous part of the Polzberg section. These important palaeontological findings highlight the importance and special position within the Upper Triassic Carnian Pluvial Episode (CPE^19,20^) and the food web with its food chains of the Polzberg *Konservat-Lagerstätte*. The environmental conditions in the Reifling Basin (Fig. 1B) changed, along with the composition of the seawater, and subsequently the inhabitants of the Triassic ocean in the Austrian Alps adapted to the special conditions during the humidification of the Carnian climate in the CPE. Previous studies dealing with that transitional Julian/Tuvalian humid episode exist from numerous localities in the Northern Calcareous Alps of Austria^19^ and references therein. The CPE appears to be a worldwide phase characterized by warming and humidification (enhanced rainfall) triggered by enormous and isochronous volcanic activity at that time^20^.

In the present paper, we report all known members of the Polzberg palaeobiota, from invertebrates, vertebrates to fossilized bromalites. The study aims to present the entire Polzberg palaeobiota and to reconstruct food webs from that Carnian *Konservat-Lagerstätte*. The entirety of the fossil taxa found so far provides new insights into the Upper Triassic (lower Carnian) trophic web and food chains of the Polzberg palaeobiota.

## Geologic setting and lithology

The Upper Triassic outcrops at Polzberg (“Polzberggraben”) are located on the western slope of Mount Schindelberg (1066 m), north of the river Ois, 4 km northeast of Lunz am See in Lower Austria. Assignment of fossils and samples to the locality Schindelberg is synonymous with the locality Polzberg (= Pölzberg^11,12^; 1:50 000, geological map, sheet 71 Ybbsitz^21^, and sheet 72 Mariazell^22^; Fig. 1). The northernmost tectonic elements of the Northern Calcareous Alps (NCA) in Lower Austria are the Frankenfels Nappe, followed to the south by the Lunz Nappe. Within the Lunz Nappe in Lower Austria, the Reifling Basin^2^—an intraplatform basin during the Upper Triassic—is located between Polzberg and Großreifling. The exact position of the fossiliferous localities in the southern area of the Lunz Nappe within the lower, fossiliferous part of the Reingraben Shales was determined by GPS (global positioning system): N  47° 53′ 4.98ʺ and E  15° 4′ 28.15ʺ, market town Gaming, federal district Scheibbs.

**Figure 2:**
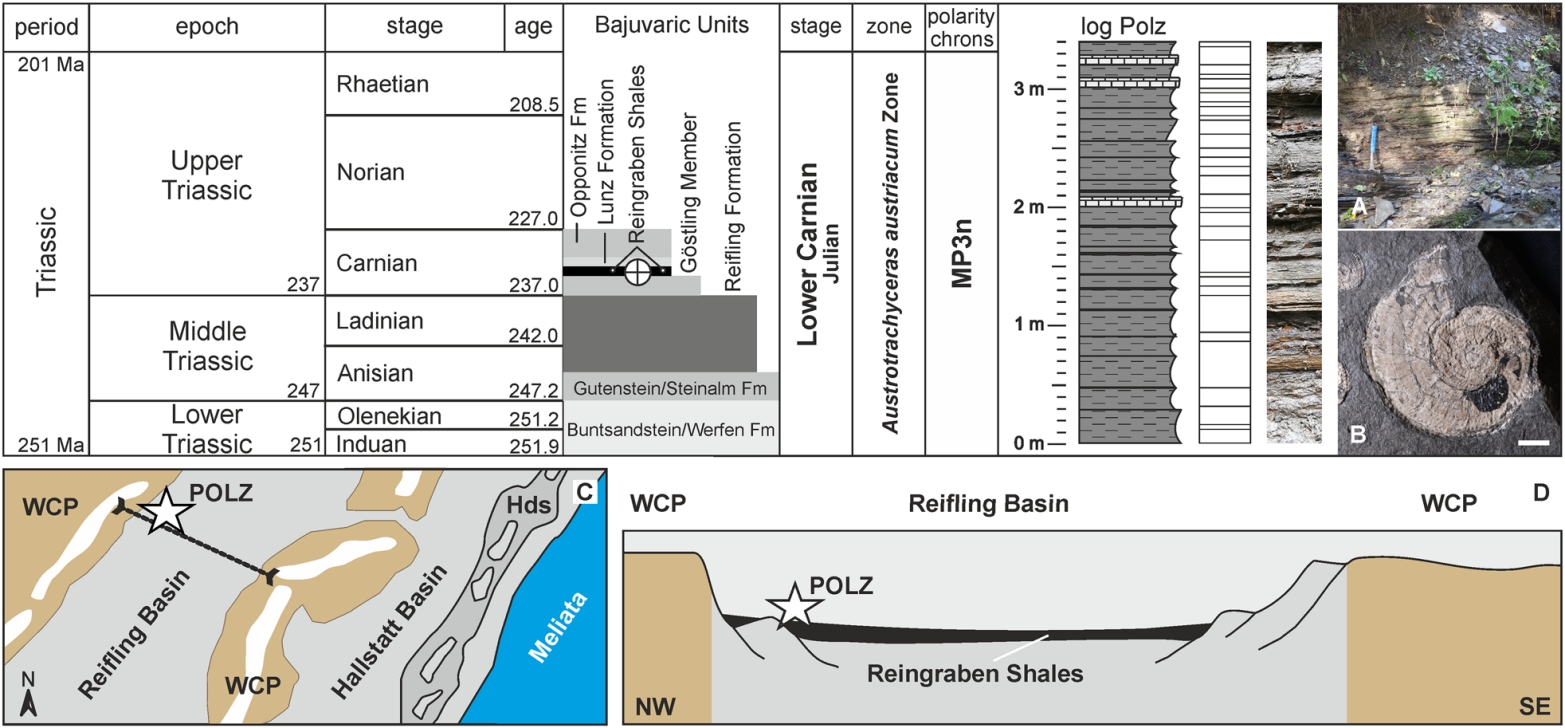
Stratigraphic position of the Reingraben Shales with the layers comprising the Upper Triassic (Lower Carnian) Polzberg palaeobiota within the *Austrotrachyceras austriacum* Zone. (**A**) Detail of the section within the Carnian Reingraben Shales at Polzberg in the ravine Polzberggraben, 16 September 2021, by PL. Images of specimens by AL. Log with indicated thickness of the Polzberg section. (**B**) Characteristic specimen of *Austrotrachyceras minor*, indicative for the Carnian biostratigraphy of the Reingraben Shales at the Polzberg *Konservat-Lagerstätte*. (**C**) Detailed palaeogeography of the Reifling Basin with the Polzberg locality POLZ, with indicated NW/SE transect of D. (**D**) Transect through the Reifling Basin with indicated position of the Polzberg locality and the deposition of the Reingraben Shales in black. WCP Wetterstein Carbonate Platform. Hds Hallstatt deep swell. Scale bar in B: 10 mm. C and D: not to scale. Prepared by AL using CorelDRAW X7; www.coreldraw.com.

Excavation campaigns to obtain the fossils were organized by the Geological Survey of Austria (GBA) in 1885 and the Natural History Museum Vienna (NHMW) in 1909. Under supervision of the mine inspector Josef Haberfelner, two adits for fossil mining were driven into the middle and basal part of the Reingraben Shales. The historical, abandoned and collapsed mines were located at N  47° 53′ 23.31ʺ and E  15° 4′ 45.80ʺ. Since 2005 the private collectors Birgitt and Karl Aschauer have sampled approx. 20 m down the stream the vicinity of the historical mine tunnels in the same fossiliferous layers. The lower Upper Triassic (*Austrotrachyceras austriacum* Zone, Julian 2, lower Carnian; approx. 233 million years^23^; Fig. 2) deposits at Polzberg are composed of Reingraben Shales (= “Aon Schichten”, “Aon Schiefer”, “Aonoides Schiefer”). These are dark grey to black claystones, marlstones and marly limestone layers and rare intercalated sandstone layers. The basal part of the Reingraben Shales, directly above the Göstling Member (Fig. 2), appears with a finely, distinctly millimetre-laminated “Ildefonso type” interval (bright/dark stratification), without bioturbation^2,3,5^. The Reingraben Shales are approx. 50 m thick and replaced at the top by deposits of the Lunz Formation with its famous Upper Triassic Lunz flora. Due to the soft nature of the marly deposits, the entire area encompassing the Reingraben Shales is brownish and weathered down to few metres at the surface, which requires mines or fresh, undisturbed outcrops at the nearby stream.

Pyrite is finely disseminated throughout the laminated, organic-rich marlstones and calcareous shales. The calcium carbonate contents (CaCO_3_ equivalents calculated from total inorganic carbon) vary between 86.9% (marly limestone) and 2.9% (claystone/mudstone), and the TOC (Total Organic Carbon; weight %)-values within the *Austrotrachyceras* abundance zone vary between 1.4 and 0.3%. The total sulphur (TS) content ranges between 1.8 and 0.3%.

The laminated appearance of the rock is a result of wispy, discontinuous, flaser-like laminae of dark, amorphous organic material and pale-coloured laminae comprising masses of halobiid shells composed of light grey to whitish calcite. The laminae and layers range in thickness from 0.1–0.2 mm to 10–25 mm. The contact surfaces between the layers and laminae are gradational to sharp. Phosphatic debris is abundant and consists mainly of actinopterygian fish scales, bones and teeth. The dominant benthic bivalves form shell pavements of juvenile to adult *Halobia rugosa*.

Reingraben Shales are considered here as an informal lithostratigraphic unit (= “Reingrabener Schiefer”^24,25^; “Halobienschiefer” ^25^; “Trachyceraten Schichten”^2,25^; = “Fischschiefer”^25^), as siliciclastic-influenced and fine-grained facies types within the Reifling Basin during the lower Upper Triassic (Fig. 2).

## The Polzberg taxa

**Figure 3:**
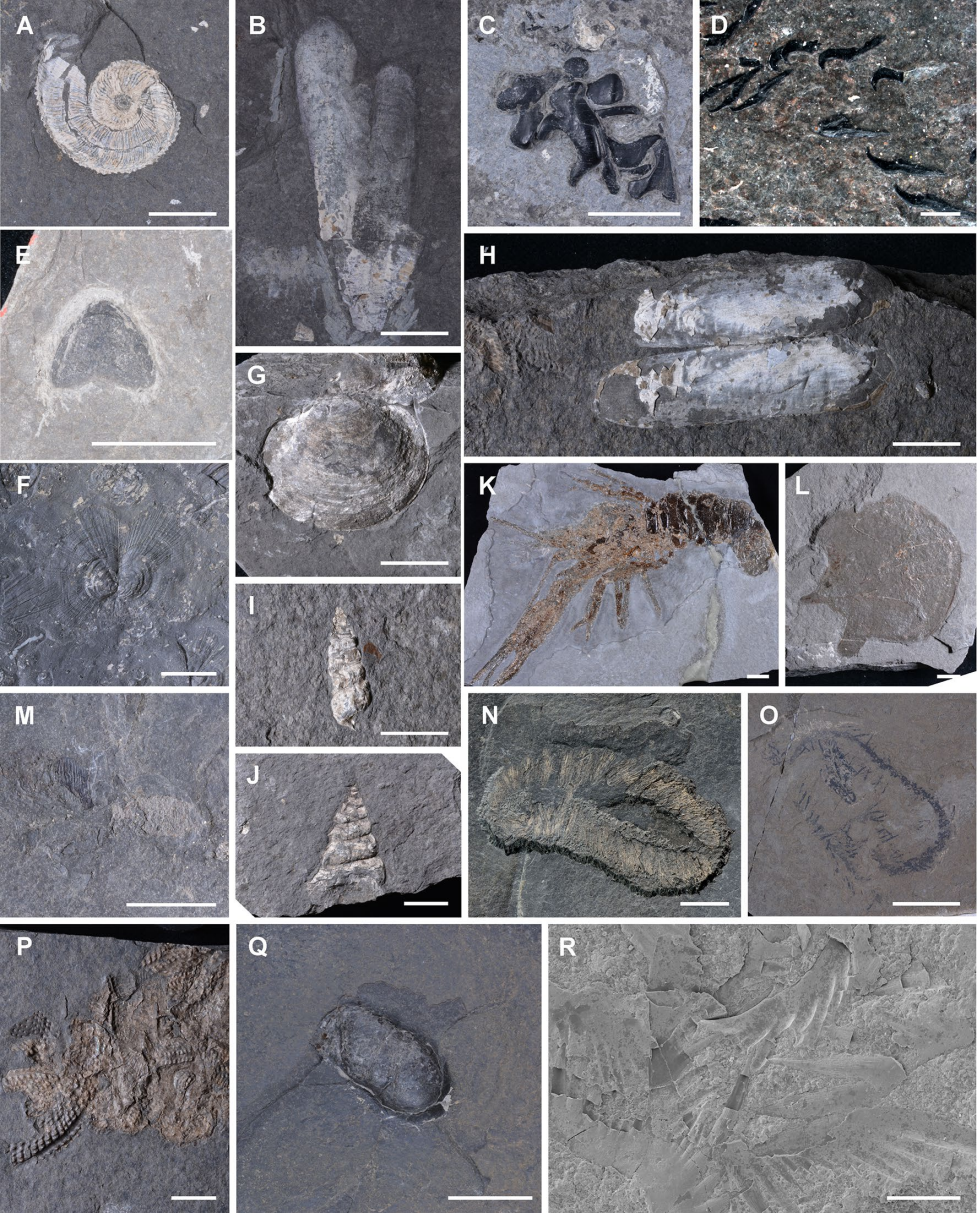
Invertebrate members of the Lower Carnian (Upper Triassic) Polzberg palaeobiota. (**A**) *Austrotrachyceras minor*, lateral view, NHMW 2021/0001/0001; (**B**) *Phragmoteuthis bisinuata*, NHMW 2006z0235/0006; (**C**) fragments of teuthid cartilage, NHMW 2021/0001/0002; (**D**) teuthid arm hooks, NHMW 2021/0001/0003; (**E**) *Anaptychus lunzensis* var. *lata*, NHMW 2021/0001/0004; (**F**) *Halobia rugosa*, note the butterfly preservation, NHMW 2021/0001/0005; (**G**) Bivalve indet, NHMW 2012/0228/0010; (**H**) Bivalve indet, note the butterfly preservation, NHMW 2012/0228/0011; (**I**) Gastropoda indet, NHMW 2012/0228/0012; (**J**) Gastropoda indet, 2012/0228/0013a; (**K**) *Platychela trauthi*, NHMW 1910/0015/0018; (**L**) *Austriocaris carinata*, NHMW 1910/0015/0050; (**M**) *Atropicaris striata*, NHMW 2021/0001/0006a; (**N**) *Palaeoaphrodite* sp.*,* NHMW 2018/0103/0003a; (**O**) eunicid polychaete, NHMW 2021/0001/0007a; (**P**) entire regurgitalite, exhibiting fragmented ammonite remains of *Austrotrachyceras minor*, ammonite fragments in external and lateral view, NHMW 2020/0033/0005; (**Q**) entire coprolite, exhibiting actinopterygian fish remains, NHMW 2020/0033/0028a; (**R**) micro-coprolite, exhibiting conodontophorid remains as cluster, NHMW 2012/0117/0023. Scale bar: 10 mm, except (**R**) scale bar: 100 μm. Prepared by AL using CorelDRAW X7; www.coreldraw.com.

**Figure 4:**
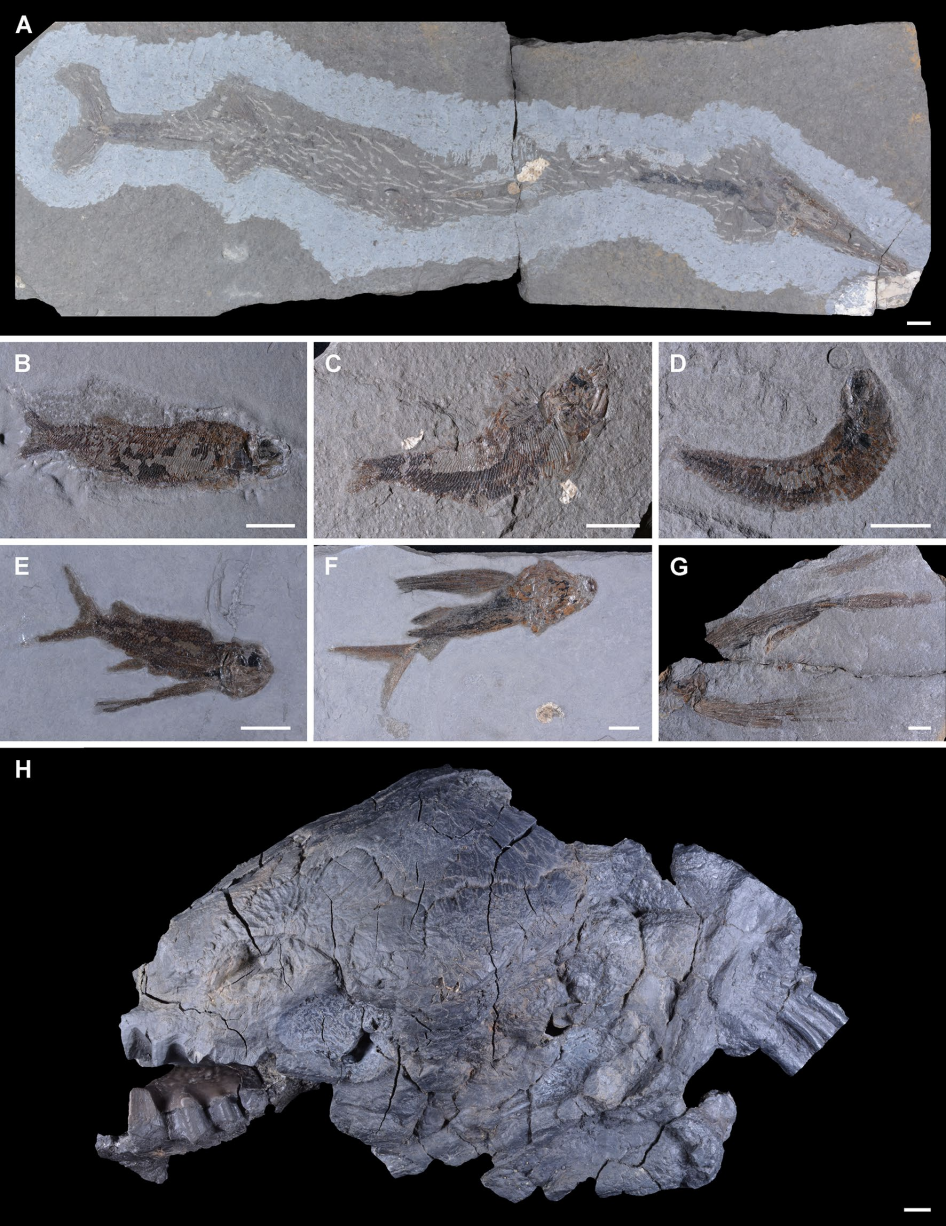
Vertebrate members of the Lower Carnian (Upper Triassic) Polzberg palaeobiota. (**A**) *Saurichthys calcaratus*, lateral view, NHMW 2007z0170/0001; (**B**) *Nannolepis elegans*, NHMW 2007z0170/0147; (**C**) *Nannolepis elegans*, NHMW 2007z0170/0148; (**D**) *Habroichthys gregarius*, NHMW 2007z0170/0071; (**E**) *Thoracopterus niederristi*, NHMW 2007z0170/0171; (**F**) *Thoracopterus niederristi*, NHMW 2007z0170/0172; (**G**) *Gigantopterus telleri*, NHMW 2007z0170/0366; (**H**) *Tellerodus sturii*, cast of GBA 1891/001/0001. Scale bar: 10 mm. Prepared by AL using CorelDRAW X7; www.coreldraw.com.

**Figure 5:**
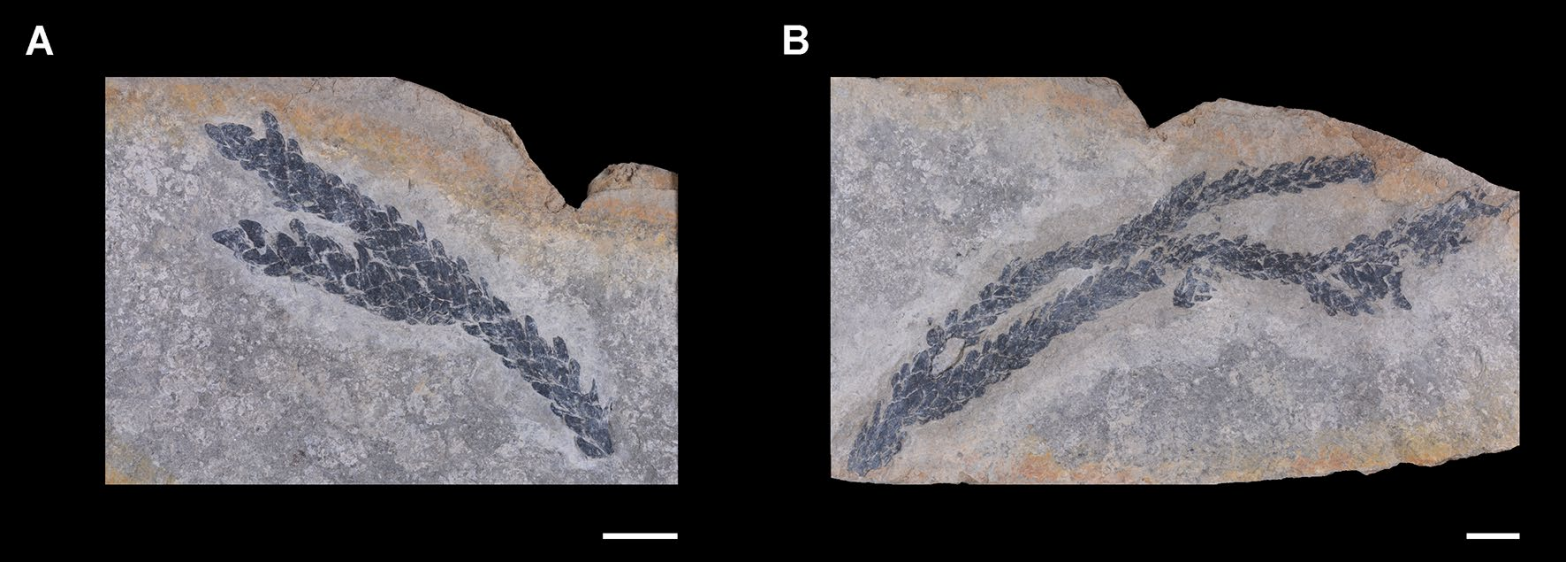
Plant members of the Lower Carnian (Upper Triassic) Polzberg palaeobiota. (**A**) *Voltzia* sp., GBA 20212007z0170/0001; (**B**) *Voltzia* sp., GBA 2021/0170/0147. Scale bar: 10 mm. Prepared by AL using CorelDRAW X7; www.coreldraw.com.

Around 1885 and 1909, thousands of fossils were collected from the Polzberg locality during the excavation campaigns of the GBA and the NHMW^12,13^. The Upper Triassic Fossil-*Konservat-Lagerstätte* Polzberg, with deposits of black, finely laminated Reingraben Shales, is poorly described and even less well understood. Stur^11^ and Teller^13^ were pioneers for the Polzberg area and its fauna by publishing preliminary data on the Polzberg outcrops. Stur^11^ erroneously termed the actually Upper Triassic (Carnian) deposits as Middle Triassic “Wengerschiefer” (= “Wengener Schichten” or Wengen Formation; Ladinian in the Southern Alps) and reported frequent *Ammonites aon* and the coleoid *Acanthotheutis bisinuata*, accompanied by the bivalve *Halobia*, along with the crustacean “clam shrimp” *Eustheria* and one actinopterygian fish *Belonorhynchus striolatus*. Thirty-seven fossil marine taxa (genera) are distinguished within the Polzberg palaeobiota 6397 specimens from invertebrates to vertebrates are recorded, and more specimens are being found every excavation season. The enormous amount and the quality of the varying fossil taxa enables special insights into the morphology of such otherwise rarely preserved fossil taxa. The Polzberg palaeobiota shows a nekton-dominated fauna with abundant fishes and cephalopods^5^. The main faunal elements (Figs. 3, 4, 5) are the bivalve species *Halobia rugosa* and ammonites of the ceratid species *Austrotrachyceras minor*^2^ (= *Trachyceras triadicum* var. *minor*^26,27^ CLXXXVI = 186, p. 682). Constituents are ammonites^2,5,26,27^ (n 4522), anaptychi^28^ (n 46), coleoids^16,17,29^ (proostraca, phragmocones, hooks, cartilage; n 386), bivalves^2,11,30^ (n > 10.000), gastropods^30^ (n 96), arthropods^3,18,30^ (n 207), polychaetes (n 17), echinoderms^30^ (n 1), trace fossils^5^ (bromalites; n 112), conodontophorids (n 12), fish^5,11,14,31–33^ (n 1181), chondrichtyes^2,5^ (n 1); lungfish^12,13,34,35^ (skull with attached teeth plates; n 1), coelocanths^13,36,37^ (n 5) and rare plant remains (n 12; Table 1). *A. minor* appears with partly preserved buccal apparatuses of anaptychus-type lower jaws^28^. In numerous specimens of *Phragmoteuthis bisinuata*^16,17^ the tripartite proostracum and the phragmocones appear with black bituminous sheets of the ink sac, along with black amorphous cartilage and arm hook structures. *Halobia rugosa* (1–30 mm length) appears mostly in double-valved butterfly preservation. The deposits of the Reingraben Shales at Polzberg are scarce in microfossils or lack them entirely. The main Polzberg collections are housed at the NHMW and the GBA.

**Table 1:** Showing the taxa from the Carnian Polzberg palaeobiota.

	Taxa/species	Reference/Fig	Ecology/mode of life	Locality Polzberg
**Invertebrata**				
Ammonoidea	*Austrotrachyceras minor*	^2,26,27^ 3A	Normal marine/nektic carnivorous, scavengers	Historic mine tunnel and new locality
	*Austrotrachyceras haberfellneri*			
	*Carnites floridus*			
	*Sageceras haidingeri*			
	*Simonyceras simonyi*			
	Anaptachus lunzensis	^28^, 3E		
Coleoidea	*Phragmoteuthis bisinuata*	^16,17^ 3B–D	Normal marine/nektic carnivorous, scavengers	Historic mine tunnel and new locality
	*Lunzoteuthis schindelbergensis*	^29^		
Bivalvia	*Halobia rugosa*	^2,11^ 3F	Normal marine/benthic filtering	Historic mine tunnel and new locality
	bivalves indet	^30^; 3G,H		
Gastropoda	caenogastropod or heterobranchs	^30^ 3I,J	Normal marine/benthic grazing	Historic mine tunnel and new locality
Arthropoda	*Austriocaris carinata* *Atropicaris striata*	^3,30^; 3L ^3,30^; 3M	Normal marine/benthic grazing scavengers	Historic mine tunnel and new locality
	*Platychela trauthi*	^30,31^; 3K		
	*Antrimpos* sp.	^31^		
	decapod lobster			
	*Obtusotelson summesbergeri* *Discosalaputium aschauerorum*	^18^		
	*Eustheria minuta*	^11^		
Polychaeta	*Palaeoaphrodite* sp.	3N	Normal marine/benthic microphagous scavengers	Historic mine tunnel and new locality
	*Eunicidae* indet	3O		
Echinoidea	Echinoidea indet	^30^	Normal marine/benthic grazing	Historic mine tunnel and new locality
Trace fossils	coprolites	^5^; 3Q	Normal marine/benthic	Historic mine tunnel and new locality
	regurgitalites	^5^; 3P		
**Vertebrata**				
Actinopterygii	*Saurichthys calcaratus*	^2,5,14^; 4A	Normal marine/nektic gregarious predatory carnivorous herbivorous	Historic mine tunnel and new locality
	*Polzbergia brochatus*, *Peltopleurus dirumptus*	^2,14^		
	*Nannolepis elegans*,	^14^, 4B,C		
	*Habroichthys gregarius*	^2,5,14^; 4D		
	*Pholidophorus latiusculcus* *Phaidrosoma lunzensis* *Elpistoichthys pectinatus* *Elpistoichthys striolatus* *Pholidophoretes salvus*	^14^		
	*Thoracopterus niederristi*	^2,5,14,32,33^; 4E,F		
	*Gigantopterus telleri*,	^14,32^; 4G		
	*Semionotus* sp.	^14^		
	Other palaeoniscids	^14^		
Sarcopterygii	*Tellerodus sturii*	^12,13,34,35^; 4H	Freshwater environments/nektic normal marine omnivorous	Historic mine tunnel
	*“Coelacanthus” lunzensis*	^2,13,36^ ^37,38^		
Chondrichthyes	*Acrodus* sp.	^2,5^	Normal marine/nektic carnivorous	Historic mine tunnel
Conodontophorida	*Mosherella* sp.	3R	Normal marine/nektic carnivorous	Historic mine tunnel
**Plants**				
Coniferopsida	*Voltzia foetterlei*	5A,B	Terrestric	Historic mine tunnel and new locality

Indicated are the taxonomic groups, the genera/species; ref/fig consecutive numbers to references and figures in the main text and reference list. Ecology and mode of life are given with information to the detailed locality historic or recent situation.

## Biostratigraphy: the *Austrotrachyceras minor* abundance zone

The *Austrotrachyceras minor* abundance zone is bordered by biohorizons which are characterized by a sharp and significant biostratigraphic change within the fossil assemblage and/or a change in the frequency of its members, as observed at Polzberg^2,39^. The lower Carnian fossiliferous deposits at Polzberg appear to be deposited during the Julian 2 Ib (*Austrotrachyceras austriacum* Zone, *Austrotrachyceras minor* biohorizon). The *Austrotrachyceras minor* biohorizon is underlain by the *A. triadicum* biohorizon and overlain by the *Neoprotrachyceras oedipus* Subzone with the basal *Austrotrachyceras* n. sp. 1 biohorizon^40^. Such biohorizons are very important for lateral correlations. The presence of abundance zones (“ammonite beds”; characterized by abundance or mass-occurrence of ammonites) is exceptionally valuable for the interregional correlation of the Late Triassic. Such uniformity beds are formed by a monotonous ammonite assemblage from at least a single bed up to few metres thickness. The appearance of the abundant index ammonite *A. minor* within the fossiliferous interval is crucial for the understanding of the biostratigraphical and interregional linkage of the lower Carnian (Julian) Polzberg *Konservat-Lagerstätte*.

## The Polzberg *Konservat-Lagerstätte* linked to the Carnian Pluvial Episode

During the Carnian (Late Triassic), the Polzberg area was located at the north-western rim of the Tethys in an area of 15° N to 30° N^19,40^ (Figs. 1B, 2B). The dry Middle and Upper Triassic climate was interrupted by a middle Carnian global phase of increased humidity in the western Tethys and hence in Europe. This episode is characterised by a worldwide decrease of platform inhabitants and reef demise known as the Carnian Pluvial/Humid Episode (CPE^19,20,41–43^). The episode was a longer and multi-phased process rather than a single event^44^. This humid phase was also termed the “Middle Carnian Pluvial Event”^45^ and is characterized by abundant siliciclastics transported by large rivers from the Baltic Craton towards the north-western branch of the Tethys. The sudden increase in siliciclastic input explains the breakdown of the carbonate factory^45^. The “Reingraben turning point”^46^ is reflected in biofacies, lithofacies, and in evolutionary events and is mirrored in all facies belts of the entire NW Tethyan continental margin^45^. The humidification is reflected by a change in lithology and facies. The basal Julian sequence in the Polzberg area is characterized by nodular limestones of the Reifling Formation deposited on the palaeoslope. At the base of the Julian 2 *Austrotrachyceras austriacum* Zone (*A. austriacum* Subzone, biohorizon of *A. triadicum*), the Reifling Formation is replaced by the limestone deposits with organic-rich mudstones of the Göstling Member to terrigenous siliciclastic deposits of the Reingraben Formation. Global warming, combined with enhanced humidification during the Early Carnian, and the eruption of large amounts of volcanogenic material, probably triggered a climate change during that time interval^39,43,45,47,48^. Recent investigations on palynomorphs by^39^ support a general trend from a dry climate in the Julian 1 to more humid and warmer conditions during the early Julian 2, corresponding to the deposition of the Reingraben Formation.

## The palaeoenvironment of the Carnian Polzberg deposits

The laminated deposits of the Reingraben Shales were formed in a relatively deep marine environment within an intra-platform basin, as inferred from the dominance of a nektonic fauna^3,5^ and references therein,^14^. The well-preserved soft bodied fauna (carbonisation, phosphatisation), the abundance of organic material in the sediment, the presence of common framboidal pyrite crystals, the absence of sessile organisms, and the lack of bioturbation point to dysoxic to anoxic bottom conditions during the deposition of the Reingraben Shales^5,30^. The Polzberg sub-basin within the Reifling Basin was mainly normal marine with ephemeral and limited freshwater input^14^. Low energy on the sea floor (absence of bottom currents) and dysaerobic conditions, which prevented predators from separating the ammonite shells from the jaw apparatuses, led to the extraordinary preservation of the Polzberg palaeobiota with entire fish carcasses, fragile taxa, ammonite conch-jaw association, and abundant double-valved bivalves. These exceptional preservational features of articulated hard parts and soft body preservation are typical for *Konservat-Lagerstätten*^1^. Bottom-water dysoxia-anoxia is known to be connected to increased levels of fossil preservation, for example as articulated hard parts of multi-element skeletons such as arthropods, echinoderms and vertebrates or in the form of preserved soft tissues. When oxygen concentrations drop below a critical threshold level (0.1 ml/l dissolved oxygen), bioturbation virtually ceases and laminated, organic-rich deposits accumulate^49^ as observed in Polzberg with the Reingraben Shales.

Subsequently, oxygen-related changes in benthic and endobenthic layers yield different communities and ichnocoenoses. The absence of bioturbation in the dark-laminated layers of Polzberg within the Reifling Basin appears to be controlled by oxygen conditions in the substrate. Oxygen availability was highly variable during the deposition of the Carnian sediments of this basin, depending on the climate changes and subsequent adaptation of the palaeoenvironments in the Polzberg area. Species abundance is a simple feature to measure relative palaeo-oxygen levels. Thus, the non-genetic, oxygen-restricted biofacies (ORB) scheme has been proposed^50^. ORBs are defined simply by their number of species and the sediment fabric. ORB 3 and 4 contain only a few benthic species which can be either very (ORB 3) or prolifically abundant on some bedding planes (ORB 4^50^), as observed at Polzberg with *Halobia rugosa* mass occurrences. The situation within the shales here fits best with an ORB 4 biofacies. Under totally anoxic conditions, trace fossils are absent or rare^51^. The position of the redox boundary fluctuated. Oxygen concentrations changed and the duration of oxygenated phases varied. Short-term low oxygenated conditions with low oxygen values in the bottom water favoured the colonialization of monotonous but abundant benthic epifauna. There were long phases with opportunistic benthic taxa or no benthic fauna at all, where the nekton such as fish and ammonoids dominated the macrofauna (i.e. no oxygen near the sea floor).

No sorting due to sedimentological or biological effects is visible; fossil alignments or concentrations triggered by bottom current transport are lacking in the Polzberg assemblage. An enrichment by redeposition by currents or turbidites can be clearly ruled out based on the autochthonous character of the nearly monospecific benthic macrofauna with its dominant element, the thin-shelled halobiid bivalve *Halobia rugosa*. Except for the transported skull of the freshwater inhabitant *Tellerodus* and of terrestrial plant material (*Voltzia*), the autochthonous character of the Polzberg deposits is strengthened by the preservation of fragile parts and the extraordinary preservation of in situ buccal (anaptychi) associations within or close to the body chambers of *Austrotraychyceras minor*. The geochemical results, along with the laminated fabric and facies, abundant organic matter and high amounts of sulphur indicate that the assemblage was deposited under conditions of intermittent oxygen depletion associated with stable water masses. A dynamic environment, controlled by short- and long-term fluctuations in oxygen levels, along with poor circulation of bottom-water currents within an isolated, basin-like region, led to the accumulation of the *Austrotrachyceras* abundance zone (= “*Trachyceras* Schichten”). The lamination generally indicates a very quiet depositional environment undisturbed by currents. Within the Reingraben Shales, dysaerobic (not anaerobic^49–51^) conditions prevailed, allowing endobenthic colonization of the incompletely bioturbated sediment. Decreasing levels of dissolved oxygen in bottom waters over time are suggested by thin, black, laminated limestones ('black shales'). The *Austrotraychyceras* abundance zone is situated in the laminated deposits. The following features are observable: (I) high TOC, (2) high sulphur content, (3) concentrations of pyrite, (4) phosphatic coprolite structures, (5) distinct lamination, (6) absence of trace fossil community (7) entire fish remains, (8) almost monospecific benthos (e.g. halobiids), (9) rare microfauna, (10) “mass-mortality” of *Austrotrachyceras*, (11) nearly 'monospecific' faunal spectrum caused by dominance of one element and (12) in situ anaptychi.

The above features characterize the shales and the incorporated palaeobiota of the Polzberg *Konservat-Lagerstätte*, deposited during the Carnian Pluvial Episode^52^ or Carnian Wet Intermezzo^53^. The sequence points to the deposition in warmer and wetter conditions, followed by a carbonate plattform decline in the Carnian world, which triggered new environmental conditions in marine and terrestrial regions.

### The Polzberg palaeobiota

**Figure 6:**
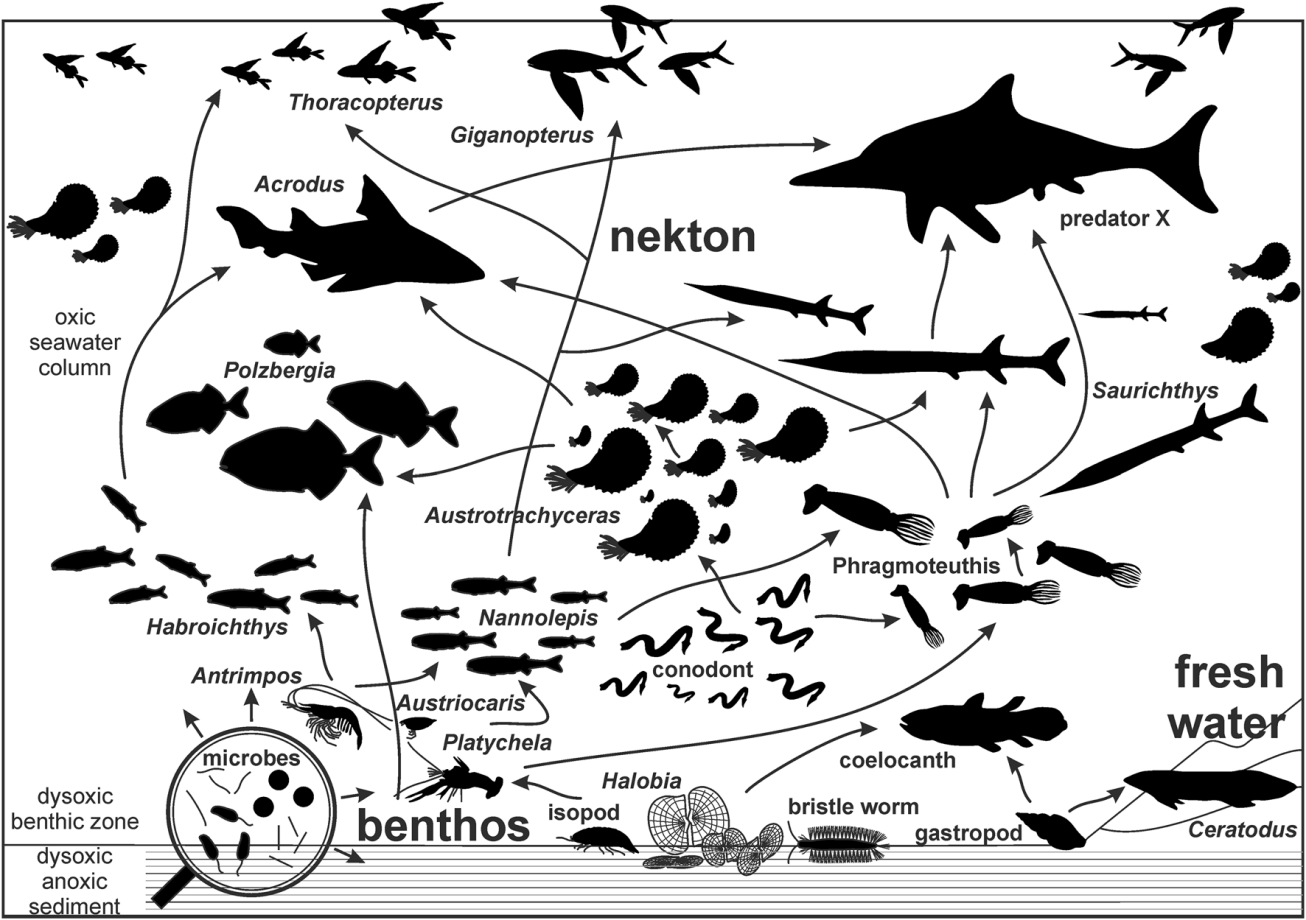
Hypothesized trophic food web of the Carnian Polzberg palaeobiota based on direct and indirect evidence from the fossil record of the Polzberg *Konservat-Lagerstätte*. Direct evidence from bromalites, indirect evidence as generalized interpretation of occurring members within the Polzberg assemblage. Figured members of palaeobiota not to scale. Artwork prepared by AL using CorelDRAW X7; www.coreldraw.com.

Members of the diverse invertebrate assemblage appear sporadically throughout the section with ammonites (*Austrotrachyceras*, *Carnites*, *Sageceras*, *Simonyceras*), coleoids (*Phragmoteuthis*, *Lunzoteuthis*), bivalves (*Halobia*), gastropods (indet sp.), thylacocephalan arthropods (*Austriocaris*), crustaceans (*Platychela*), eustheriids (*Eustheria*); isopods (*Obtusotelson*, *Discosalaputium*), and polychaetes (*Palaeoaphrodite*, Eunicidae indet). Vertebrate taxa are represented by frequent and dispersed acytinopterygid fishes (not accumulated in single layers) throughout the section (*Saurichthys*, *Polzbergia, Peltopleurus, Habroichthys*). Other taxa include remnants of cartilaginous fishes (*Acrodus*), several coelocanthid fishes (“*Coelacanthus*”), the lungfish *Tellerodus*, and a conodont cluster (*Mosherella*, Fig. 6).

Such excellent preservational deposits (i.e. *Konservat-Lagerstätten*) are rare in the European Triassic marine record. Only few comparable sites are known from Seefeld in Tyrol^9^ or Wiestal in Salzburg^10^, both within the Norian Seefeld Formation, and from the Middle Triassic (late Anisian to Ladinian) *Konservat-Lagerstätte* of Monte San Giorgio (Ticino, Switzerland^7,52^). Special conditions are required for the formation of such conservational deposits with fragile and well-preserved fossil remains. Stagnation of water masses along with terrigenous influx by enhanced runoff resulting in accumulations of organic material led to dark-laminated, pyrite-rich deposits that promoted the soft tissue preservation of fishes^54^ and other fossil taxa. The Polzberg palaeobiota was deposited in an intraplatform basin, which intensified these conditions, as the Reifling Basin was surrounded by the Wetterstein platform. The demise of platforms with a co-occurring carbonate breakdown was a worldwide phenomenon at that time. This promoted the sedimentation of argillaceous sediments, and the shale deposits were influenced by the enhanced runoff from emerged land, representing former shallow submarine platforms. The enhanced run-off was triggered by the increasd humidity during the Carnian Pluvial Phase (CPP;^40,42–44^). The Carnian was characterized by a worldwide humidification in the Carnian Pluvial Episode and by a sea level regression. Both events enhanced the stratification effect in the newly formed intraplatform basins. This also helps to explain the origin of fossil remains in such distinct Fossil Lagerstätten comprising a variety of marine and freshwater taxa accompanied by plant remains. The preservation of a palaeocommunity including benthic (epifaunal and infaunal) and nektonic taxa point to a deposition within the inhabited palaeohabitat where the organisms primarily lived, with minimal post-mortem drift or transport. Interestingly, the occurrence does not show densely packed ammonoid shells as expected for sudden-death events in which fossils are preserved in accumulations or mass-occurrences. Such mass mortality events are preserved in Wiestal, where masses of fishes are accumulated in very thin layers (i.e., mm thickness, 5 fish layers^10^). Nektonic fish remains and ammonite shells in the host rock do not exhibit any size sorting (shell diameter from 4 to 80 mm) and lack preferential orientation by bottom water currents. Taphonomic evidence suggests that the Polzberg palaeobiota was formed by stagnant, oxygen-depleted basinal waters without major transport or reorientation of fossil carbonate shell material or fish carcasses.

The main diagnostic criteria for the presence of a *Konservat-Lagerstätte* are fulfilled^3,5,14,16^. More specifically, such unique windows into Earth history contain entire fossil remains, grouped fossil parts, in situ preservation, soft tissue preservation and/or normally rarely preserved fossil remains of numerous fragile fossil taxa. Such palaeocommunities mirror the trophic conditions of the palaeo-food web at the time of deposition (Fig. 6). Fossil remains are not significantly affected by benthic scavengers or bacterial decay. Contrastingly, frequent shell fragments, crushed by nektonic predators, and well-preserved bromalites are main but little-known constituents of the fossil record in the Polzberg palaeobiota^5^. Bromalites consist mostly of fish coprolites and rare regurgitalites. The evidence suggests that Polzberg locality preserves two types of bromalites: coprolites incorporating fish remains with fish scales, and regurgitates with ammonites and coleoid hooks and cartilage masses. Additional recent findings show bromalites with only one constituent, i.e. dominated either by hooks from teuthids, ammonite shells or fish scales. The predators here were therefore apparently specialized on different diet strategies and prey. Most likely, different actinopterygiid fishes equipped with various dentitions fed on cephalopods or other fishes. Additional feeding types occurred at the sea floor in the form of scavengers, harvesting organisms or decomposition of organic metarial.

A single specimen of the lungfish *Tellerodus sturii* was also found here^26^. Mesozoic dipnoans were restricted to freshwater environments and their remains found in marine deposits are commonly interpreted as a result of post mortem transport from freshwater ecosystems. Conchostraca appear frequently with *Eustheria* in the upper, more argillaceous part of the Polzberg section. Eustheriids typically inhabit freshwater or at least brackish environments. Both elements—the vertebrate lungfish *Tellerodus* and the conchostracan shells—indicate a sporadic influx of freshwater or sedimentation from surrounding shallow-water or terrestrial areas into the restricted Reifling Basin (incorporating the Polzberg zone). A possible adaptation of these new cocnchostracan species to marine environments cannot fully be excluded but requires more detailed investigations on their distribution based on bed-by-bed sampling. Plant remains with foliated trunks of the Coniferophyta member *Voltzia* support this interpretation.

### Food web of the Polzberg palaeobiota

**Figure 7:**
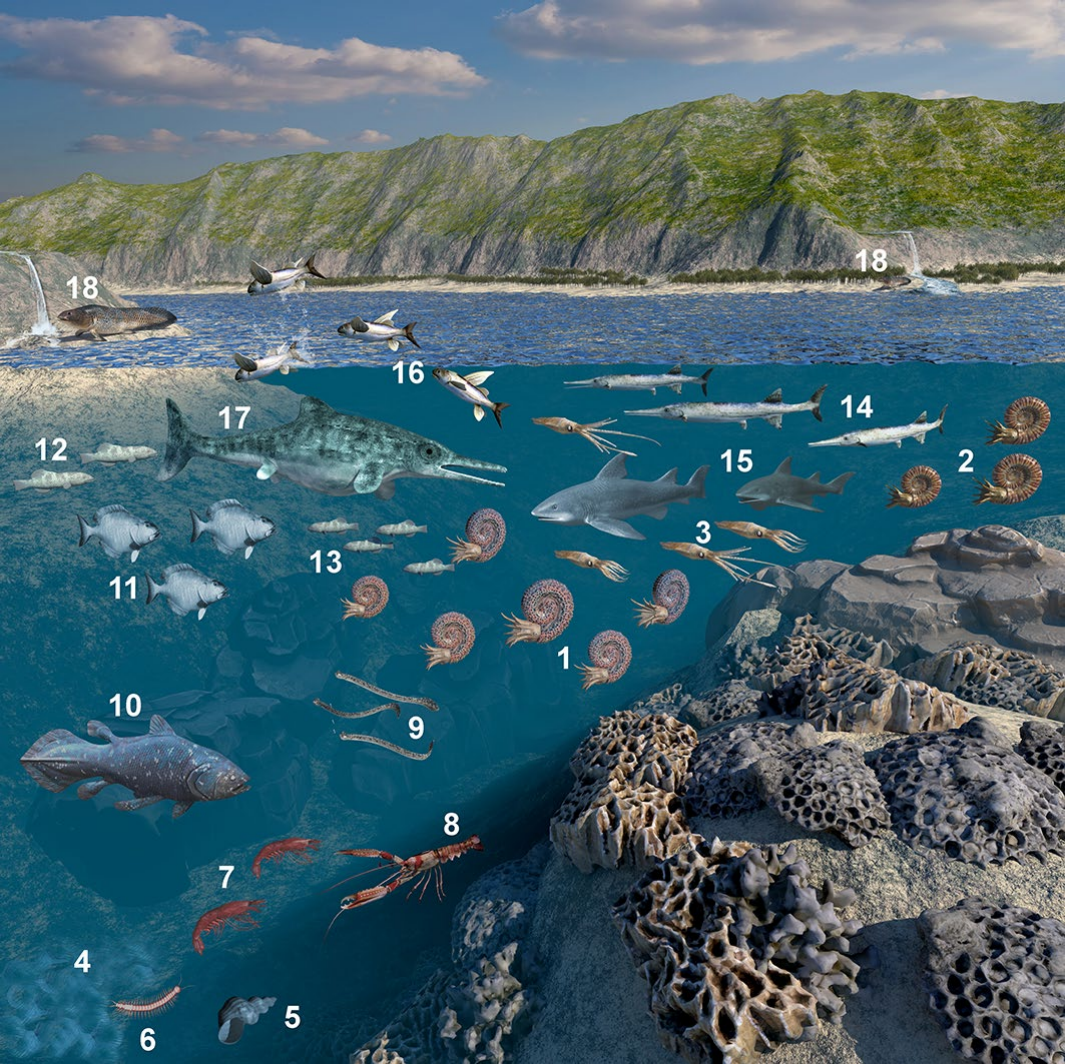
Reconstruction of the palaeoenvironment and members of the Carnian Polzberg palaeobiota. **1** *Austrotrachyceras minor*, **2** *Austrotrachyceras haberfellneri*, **3** *Phragmoteuthis bisinuata*, **4** *Halobia rugosa*, **5** caenogastropod, **6** *Palaeoaphrodite*, **7** *Antrimpos*, **8** *Platychela trauthi*, **9** conodontophorida, **10** *“Coelacanthus” lunzensis*, **11** *Polzbergia brochatus*, **12** *Habroichthys gregarius*, **13** *Nannolepis elegans*, **14** *Saurichthys calcaratus*, **15** *Acrodus*, **16** *Thoracopterus niederristi*, **17** predator X, **18** *Tellerodus sturii*. Figured members of palaeobiota not to scale. Artwork based on Fig. 5 by AL using CorelDRAW X7; www.coreldraw.com. Final artwork by 7reasons; www.7reasons.net.

Over the last 140 years, 6397 fossils have been collected here during several excavation campaigns and by citizen scientists. The amount and variety of fossil remainsenable conclusions on the palaeo-food web of the Upper Triassic Polzberg deposits (Fig. 6), dominated by benthic halobiid bivalves, nektonic actinopterygiid fishes and ceratitid ammonites^5^ (Fig. 7).

The primary producers in this food web are represented by algae and bacteria, grazed by primary consumers such as gastropods and arthropods and filtered by benthic bivalves. The low-level consumers are preyed upon by secondary consumers or predators including different actinopterygiid fishes and ammonites. The latter groups served as prey for larger cartilaginous fish (*Acrodus*) and the actinopterygiid fish *Saurichthys*. The presence of a top predator of the ichthyosaur group is speculative but probable. Thylacocephalan arthropods, represented by *Austriocaris*, are thought to have had either a scavenging mode of life near the sea floor, as shown for *Ostenocaris*^55,56^, or as actively hunted in the water column for conodonts, small cephalopods^57^ and other small members of the palaeobiota see also^57^. *Ostenocaris* was found in the lower Jurassic of Osteno (Italy) with stomach contents or regurgitalites comprising fish vertebrae or scales and coleoid arm hooks^55,56^. Late Devonian thylacocephalans from Maïder (Morocco) with *Concavicaris* were assigned as predatory carnivors^57^, as also shown for *Dollocaris*, a middle Jurassic thylacocephalan from La Voutle (France) preying on other arthropods^59^. They, in turn, were hunted themselves by chondrichthyans or other large fishes^57^, hence serving as an important food source for numerous fish taxa.

Starting at the base of the trophic pyramid at the upper Triassic sea floor from Polzberg basin, and extending across the entire food web, this system is similar to others from the Permian to Triassic marine palaeoworld, with comparable trophic levels of invertebrate and vertebrate members^10,52,58–61^.

Important evidence for food webs here is gained from bromalites, represented by coprolites and regurgitalites^5^ and references therein. As reported by Lukeneder et al.^5^, regurgitalites were produced by large durophagous predators. The cephalopods and arthropods here appear to be too small to produce bromalites up to 100 mm in size. The rich ichthyofauna and lack of reptile remains point to probable bromalite producers among predatory fishes. There is evidence that Palaeozoic and Mesozoic shelled cephalopods were preyed upon by sharks and actinopterygian fishes^41,43–45^. Krystyn^2^ and Lukeneder et al.^5^ noted the occurrence of the cartilaginous fish *Acrodus along with* the actinopterygiid predators *Elpistoichthys*, *Gigantopterus*, *Saurichthys, Thoracopterus, Habroichthys*, *Nannolepis* and *Peltopleurus*. Griffith^14^ stated that the Upper Triassic ichthyofauna of the Polzberg region is characterized by abundant flying fish, which, according to that author, suggests strong predation pressure in this marine ecosystem. Furthermore, 55% of the genera of marine fish known from Polzberg were predatory^5,14^. The largest specimens described by Griffith^14^ belonged to *Saurichthys* (50 cm in length). *Saurichthys* is an ambush predator^62^ targeting other actinopterygiid fishes, was also shown in the Norian fish assemblages from Wiestal in Salzburg^10^. Hornung et al.^10^ provided a figure of the gastric residual content of *Saurichthys deperditus* including the teeth of the neoperygiid *Paralepidotus ornatus*; another specimen contained *Pholidophorus*.

Typically, the rerurgitalites from Polzberg show spezialization of the producer to a cephalopod prey because they consist of ammonite shells, coleoids hooks and cartilage material. Ammonite shell fragments and entire shells are solely from the genus *Austrotrachyceras minor*, teuthid fragments exclusively from *Phragmoteuthis bisinuata*. No sublethal injuries are reported on ammonite or coleoid specimens here—only crushed and fragmented cephalopods pointing to immediate death by predators specialized on nektic cephalopods. In contrast the coleoid *Phragmoteuthis* could have fed on actinopteyigiid fishes and hunted small and slow austrotrachyceratids, as reported from Jurassic stomach contents and coprolites of teuthids^63^.

Additional evidence for actinopterygid fish predation on coleoids of *Phragmoteuthis* is available from the Lower Jurassic Posidonia Shale of Germany^64^. The same deposits yielded evidence for the predation of coleoids on other coleoids (Klug et al.^65^).

The marine predatory vertebrates in Polzberg that potentially produced the regurgitalites desribed herein are *Acrodus* and *Saurichthys*^5^. Durophagy sensu lato (the ability to consume hard prey^5^ and references therein) is possible with numerous dental types, especially when dealing with thin-shelled prey such as the small ammonites in the present regurgitalites^5^. We assume that various durophagous actinopterygiids hunted and crushed their prey, including *Elpistoichthys*, *Gigantopterus*, *Saurichthys, Thoracopterus, Habroichthys*, *Nannolepis*, and *Peltopleurus*. Triassic species of *Saurichthys* are characterized by monognathic heterodonty—the teeth in one jaw differ in size and shape. Given the above, we argue that a durophagous shark such as *Acrodus*, which was equipped with a typical durophagous dentition (crushing or grinding) with blunt and broad teeth, most likely produced the studied regurgitalites^5^ As noted by Lukeneder et al.^5^, the more abundant but smaller and longitudinal coprolites, containing masses of almost exclusively fish scales, were most likely produced by medium-sized piscivorous actinopterygians common in the Polzberg palaeobiota: *Elpistoichthys*, *Gigantopterus*, *Saurichthys, Thoracopterus, Habroichthys*, *Nannolepis*, and *Peltopleurus*.

No evidence is currently available of a possible top “predator x” (Fig. 7) from the late Triassic ichthyosaur- or nothosaur-group see^66–69^ in the Polzberg basin, but fossil finds are expected in upcoming excavation campaigns. This would enable testing the top predator hypothesis.

## Conclusions

This is the first report on the discovery of historical and recent findings of the palaeobiota from the Polzberg *Konservat-Lagerstätte* to a broader, international scientific community. The Upper Triassic (Carnian) Polzberg locality from the Austrian Alps yieded producers, consumers, as well as small and large predators within the frame of the Reifling intraplatform basin during the Carnian Pluvial Episode (CPE). This worldwide humidification in the Carnian caused the deposition of the dysoxic sediments of the Reingraben Shales at the epipelagic to upper mesopelagic sea floor of the basin, which was periodically disconnected from oxygenated bottom currents. In the low-oxygen ecosystems at Polzberg, bivalves of the genus *Halobia* were the dominant epifaunal elements, at least near the sea floor and/or within the carbon-rich and laminated sediment. In the overlying oxygenated water column, ceratitid nektonic/nektobenthic ammonites (*Austrotrachyceras*) and nektonic actinopterygiid fishes prevailed. The occasional freshwater influx from the surrounding Wetterstein Platform resulted in terrigenous and argillaceous sedimentation, This was accompanied by terrestrial plant material (*Voltzia*) and the freshwater dipnoid lungfish *Tellerodus*. The entire fossil assemblage provides new insights into Upper Triassic trophic interactions and the food chains of this Carnian marine ecosystem. We highlight the importance of multi-pronged analyses—taxonomy, geochemistry and palaeoecology—of such conservation “Lagerstätten” to extract the entirety of hidden information in these special deposits. The presence of fragile nektonic and benthic taxa points to unique palaeoenvironmental conditions in the Carnian dysoxic bottom water of the Reifling Basin. Triassic invertebrates (e.g., ammonites, phragmoteuthids, bivalves, gastropods, crustaceans, polychaetes) and vertebrates (actinopterygiids, sarcopterygiids, chondrichtyiids) made up the marine benthic and nektic communities. Our report underlines the diverse palaeobiota including new taxa from the Triassic Polzberg *Konservat-Lagerstätte*. Our study also confirms the presence of an isolated marine intraplatform basin. During the humid and warm Carnian Pluvial Episode, that basin was affected by enhanced freshwater influx and terrigenous input of siliciclastic sediments from the surrounding emerged platform highs. Our report marks the starting point for future descriptions of numerous taxonomic members of the Polzberg palaeobiota, both vertebrates and invertebrates. This would be an important step forward in improving our knowledge of Upper Triassic marine ecosystems. Our approach also highlights the cooperation between citizen scientists and professional researchers because private collectors have sampled the Polzberg fossil site over decades. Over the next two years, further excavations are planned, and the expected findings will no doubt shed new light on the palaeobiota of the Polzberg *Konservat-Lagerstätte* deposited during the Carnian Pluvial Episode and help test several hypotheses presented here.

## Material and methods

6397 fossil remains stem from the ravine Polzberggraben (Lunz Nappe, Northern Calcereous Alps) near Polzberg (= Schindelberggraben; or given as Polzberg locality in numerous collections), between mount Föllbaumberg (1014 m) to the west and mount Schindelberg (1066 m) to the east. The investigated fossil material is housed in the collections of the NHMW and the GBA. The material was collected over the last 140 years (field campaign GBA 1886 and NHMW 1909), with a focus over the last 10 years by the private collectors Birgitt and Karl Aschauer (both Waidhofen and der Ybbs, Lower Austria). The authors contribute to these extensive collections with their own findings over the last 20 years. The fossil remains recorded herein have been investigated with a variety of analytical tools and electronic instruments.

Macro-photographs were done with a Nikon Digital Camera, D 5200 SLR, lens Micro SX SWM MICRO 1:1 Ø52 Nikon AF-S, processed by the free graphic software tool digiCamControl version V.2.1.2.0 at the NHMW. Digital high-quality photomicrographs were taken using a Discovery.V20 Stereo Zeiss microscope. The magnifications were × 10 × 20 and × 40 in incident light mode. Data from the AxioCam MRc5 Zeiss were processed and documented using the AxioVision SE64 Rel. 4.9 imaging system at the NHMW.

Thin sections of rock samples were made in the NHMW laboratories. Samples were embedded in Araldite epoxy resin, sectioned, mounted on the microscope slides and polished with silicon carbide and aluminium oxide powders to a thickness of about 19 μm.

Sulphur (% S), total organic carbon (% TOC) and total carbonate content (% CaCO_3_) were measured at the Institute for Earth Sciences (Karl-Franzens-University, Graz, Austria). Calcium carbonate content was measured using a carbonate bomb technique. Total carbon (TC) content was measured using a LECO WR-12 analyser, and TOC content was calculated as the difference between TC and CaCO_3_, assuming that all carbonate is pure calcite.
